# Supplementary material for: Catheter ablation of atrial arrhythmias in cardiac amyloidosis: Impact on heart failure and mortality
Source: PLoS One. 2024 Apr 5;19(4):e0301753. doi: 10.1371/journal.pone.0301753 (PMC10997066; doi:10.1371/journal.pone.0301753)
Supplement: Attachment — Submitted filename: answers to the editors and reviewers comments.docx [file pone.0301753.s003.docx]

Catheter Ablation of Atrial Arrhythmias in Cardiac Amyloidosis
Impact on Heart Failure and Mortality

PLOS ONE

**PONE-D-22-29848R1**

**Details on the revisions carried out on the manuscript since its original submission**

We already resubmitted a revised version end 2022 according to the editor’s remarks and concerns and reviewer comments, including the following changes. Additional editor’s comments were answered onset 2023, which were in fact fundamentally the same ones, thus our answers were also similar. This point by point response gathers all the previously answers and comments to the editor’s / reviewers concerns.

**Answer to the Editor’s Comments:**

The aim of this study is clinically relevant. However, there are some major limitations in this study that should be addressed.

1) Small sample size: just 22 patients had AF, which is the more important event/variable to analyze. Hence, the authors should increment significant the sample size of the study, including at least 200 patients in order to avoid bias and to increment the clinically relevance of this study.

Answer : this is the biggest series of AF ablation in cardiac amyloidosis to date. Probably far less than 50 patients have been ablated worldwide, because it is absolutely not the experience of most centers. Thus collecting 200 patients is simply impossible at yet. We feel that this series is large enough to draw some conclusions and anyway will be impossible to significantly increment currently at the point the editors wished.

2) Subgroup analysis: Patients with Al-Amyloidosis and ATTR-Amyloidosis should be analyzed separately, since these subtypes of amyloidosis have significantly different outcomes and treatment.

Answer : this has been done :

- « There was no significant difference between patients with ATTR and AL CA, except more paroxysmal AF and congestive heart failure in AL CA, with a younger age and lower CHA2DS2VaSC score. » page 8 line 10
- « Recurrences were not associated with age, gender, type of CA … » page 9 line 7
- « There was no difference in recurrence, mortality or hospitalizations for heart failure between ATTR CA and AL CA. » page 9 line 18
- « All the three patients with end-stage HF leading to death (all with ATTR AF) had recurrent arrhythmias compared to .. » page 10 line 8

3) The primary endpoint of this study should be only recurrence of AF at 1 and 2 years of follow-up.

Answer : the primary endpoint was not to assess the long term success rate of ablation, but rather if this will translate in better hemodynamics and less congestive heart failure and eventually in prognosis.

This was not a study devoted to crude results of ablation and about technique and so on. Thus, the primary endpoint was to assess the long-term impact of catheter ablation for atrial arrhythmias in cardiac amyloidosis regarding hearte failure and prognosis.

Anyway, in the results, long term results of ablation are mentioned:

« At final follow-up, 10 patients (32%) were in AA and 21 (68%) remained in SR over a median follow-up of 19 months » and the survival curve (free from recurrences) also is shown. On the survival curve one can see that there was 30% recurrences at one year and 70 % at 24 months (not including redo procedures). This has been added in the figure legend. So we feel that these informations are in fact more clearly present.

**Answers to the Reviewers' comments:**

Reviewer #1: In the current paper, the authors reported their experience about the impact of catheter ablation on outcomes in patients with amyloidosis. The paper was well written in general. Please include more data about the catheter ablation procedure regarding the left atrial scarring in the study group

Answer : the paper was not dedicated to precise description of ablation procedures in CA patients, but datas on left atrial scarring in patients with atrial fibrillation has been added :

« Scar surfaces (atrial tissues with less than normal voltage) were retrieved from 3D voltage maps using Mathlab ™ program and correlated to total atrial surfaces. » page 5 line 2

« Median scar surface was 85 cm2 (IQR 70) representing 75 % of total atrial surface (IQR 36). » page 8 line 15

Reviewer #2: The authors report on outcome post catheter ablation for atrial arrhytmias (AT, Aflutter or AF) in a patient series comprising 31 patients with cardiac amyloidosi.
Cardiac amyloidosis is an infiltrative disease with poor outcome, bacause of development of advanced heart failure leadingt o death.
Although the presented case series is limited with regard to the number of patients, the study is interesting and one of the rare reports on this topic. The study demonstartes the potential for improved hemodynamics, NAHY class and BNP levels, in those CA-patients in whom sinus rhythm can be maintained by combination of cathetr ablation and antiarrhythmic drug therapy. Notably, betablocker treatment was not beneficial in this patient cohort with restrictive CMP.

Answer : the thank the reviewers for these nice comments
