# Supplementary material for: Catheter ablation of atrial arrhythmias in cardiac amyloidosis: Impact on heart failure and mortality
Source: PLoS One. 2024 Apr 5;19(4):e0301753. doi: 10.1371/journal.pone.0301753 (PMC10997066; doi:10.1371/journal.pone.0301753)
Supplement: Attachment — Submitted filename: PlosOne answers to the reviewers comments.docx [file pone.0301753.s004.docx]

PONE-D-22-29848R2

Catheter Ablation of Atrial Arrhythmias in Cardiac Amyloidosis. Impact on Heart Failure and Mortality

PLOS ONE

**1.** There are minor grammatical errors in the abstract section. In methods section: “ AA were atrial fibrillation..” this statement needs better grammatical construction. Also in this section authors mention “ Atrial common flutter” , do you mean “ Atrial flutter”

**Answer: this has been corrected: “AA subtypes included atrial fibrillation (AFib) in 22 (paroxysmal in 10 and persistent in 12), atrial flutter (AFl) in 17 and atrial tachycardia (AT) in 11 patients.” See abstract**

**2.** Its not clear looking at the abstract about what the main aims of the study are. In the methods section—may be we should explain that we compared outcomes before and after ablation etc? to assess long term efficacy of ablation etc

**Answer: aims have been more developed.**

**Aims: “This study sought to investigate the long-term efficacy and impact on HF progression of catheter ablation in patients with CA and AA.” See abstract**

**Methods: “Long-term AA recurrence rates were evaluated along with the impact of sinus rhythm (SR) maintenance on HF and mortality.” See abstract**

Throughout rest of the article, there are statements which need grammatical correction such as

**Asnwer: The manuscript has been corrected by a native English author**

  “AA undergoing ablation were AFib in 22 (71%) (paroxysmal in 10 and persistent in 12), CTI-dependent AF in 17 (55%) and AT in 11 (35%)”---- better statement----   ‘ *Among patients undergoing ablation for arrythmia, 71%  had afib, 55% had CTI-dependent afib and 11% had AT” etc*

***This has been corrected: “All 31 patients underwent catheter ablation for AA: 22 (71%) for Afib (paroxysmal in 10 and persistent in 12), 17 (55%) for CTI-dependent AF and 11 (35%) for AT” page 7 lines 7-8***

*“*A previous history of catheter ablation was present in only two cases (for AF)” –  correct grammar “ only 2 cases had a hx of catheter ablation prior to our study.

**This has been corrected: “Two patients had a history of prior catheter ablation (for AFl)” Page 7 line 10**

“When comparing before and after ablation while in SR (median 9 months later, IQR 17), creatinine and natriuretic peptides levels significantly decreased as well as NYHA class, without relevant change in weight, while left atrial dimensions and pulmonary arterial pressures, although decreased, did not significantly change (see table 2)”--   Full stop after “change in weight”. Then last statement should start ( my suggestion)

**This has been corrected also: “Over a median follow-up of 9 months (IQR 17), serum creatinine and natriuretic peptides levels significantly decreased with improvements in NYHA class in patients remaining in sinus rhythm, without a significant change in weight. There was a trend towards reductions in left atrial dimension and pulmonary arterial pressure after ablation in patients in sinus rhythm (see table 2)” page 9 lines 12-16**

may be get assistance of professional grammatical correction services

**After reviewing by an English native physician, we hope that the manuscript is now free from grammatical issues.**

**3.** How do we know that this study shows long term efficacy of ablation in CA patients?

Is ablation in CA patients better or worse than ablation in patients without CA? Whats the control population to compare with?. How do we know that history of amyloidosis interacting/or not interacting with ablation outcomes

If we compared effects of ablation between patients who had cardiac amyloidosis and patients who did not have cardiac amyloidosis: then significance of these results would have been better understood.

Can we talk about this in the discussion? May be cite articles talking about outcomes of catheter ablation in general for atrial arrythmias?

**Answer: although such comparison was not the aim of this study, a short comment about the differences between AFib ablation in CA and other settings has been added in the discussion**

**“This is in comparison to other clinical situations, where long-term success rates of Afib ablation range between 50% and 80% (28). Moreover, the positive impact of sinus rhythm maintenance with Afib ablation in heart failure is well established (29). Page 11 lines 17-20**

**Comments to the Author**

Reviewer #3: 1) NYHA Class 2 was noted in the page 7, Table 1 baseline characteristics. In Page 10, prior to ablation NYHA Class 3 was mentioned. Was baseline NYHA >2?, please clarify.

**Answer: median NHYA class was 2 in the whole population at baseline, but was 3 at baseline in the group of patients in whom comparisons could be made because remaining in sinus rhythm (mentioned in the legend of table 2).**

2) Stage of Cardiac amyloidosis, when cardiac ablation was performed

**Answer: stage of cardiac amyloid is now mentioned in table 1**

**European score (AL-CA)	II 2/9	IIIa 6/9	III b 1/9**

**NAC score (ATTR-CA)	I 5/18	II 5/18	III 8/18**

3) Page 2 -Background: This study sought to investigate the long-term impact of catheter ablation in patients with CA and AA. Page 4 sentence 2,3-The aim of this study was to explore the efficacy and impact of catheter ablation in CA complicated by AA in terms of reversal of congestive heart failure and mortality.

Please clarify in page 2 background section, is the study designed to evaluate impact on cardiological outcomes?

**Answer: this has been changed in the abstract (see answer to the editor’s comments):**

**Aims: “This study sought to investigate the long-term efficacy and impact on HF progression of catheter ablation in patients with CA and AA.” See abstract**

**Methods: “Long-term AA recurrence rates were evaluated along with the impact of sinus rhythm (SR) maintenance on HF and mortality.” See abstract**

4) Page 9 -Mortality rate 39 % (12 patients)- 3 End stage CHF ,1 Sudden Cardiac Death,1 stroke (total 5). 1 pt passed away from Covid ,1 patient passed away from unknow cause (Total 2). Another 5 patients passed away from later complication of Amyloidosis? Please clarify.

**Answer: yes 5 patients died from non-cardiac complications of amyloidosis: this bas been detailed: “while late noncardiac complications of amyloidosis were the cause of death in five patients (pneumopathy in two, degradation of general conditions in two and sepsis in one).” Page 9 lines 5-7**

5) Page 8- AV nodal ablation and ICD, have their own impact on CHF and mortality, potential confounding factor

**Answer: we agree although ICD did not modify mortality in this population, and previous AV node ablation did not succeed in treating heart failure despite controled heart rate. This has been added in the limitations and results.**

**“Presence of ICD or previous atrio-ventricular node ablation may have contributed to morbidity or mortality outcomes, however no differences in mortality between implanted and non-implanted patients were observed, and the few patients with prior atrio-ventricular node ablation manifested congestive heart failure despite ventricular rate control prior to AA ablation.” Page 13 lines 5-8**

**“There was no significant difference in mortality between ICD-implanted and non-implanted patients (25% vs 47%, p=ns)” page 9 lines 9-11**

Reviewer #4: Well written paper and good research design. I am pleasantly surprised that the researchers were able to find so many patients with Atrial tachycardia and Cardiac Amyloidosis who underwent ablation. I would have loved to see a discussion about comparing the impacts of catheter ablation in patients with cardiac amyloidosis and patients who do not have amyloidosis. This would have put the results in a better perspective. Please consider including this in the discussion portion of the paper.

**This has been done, see answer to editor’s comments above: although such comparison was not the aim of this study, a short comment about the differences between AFib ablation in CA and other settings has been added in the discussion**

**“This is in comparison to other clinical situations, where long-term success rates of Afib ablation range between 50% and 80% (28). Moreover, the positive impact of sinus rhythm maintenance with Afib ablation in heart failure is well established (29). Page 11 lines 17-20**

Reviewer #5: Authors have addressed all the prior comments by reviewers and editors and manuscript can be accepted for publication.

**We thank this reviewer**

Reviewer #6: Amyloidosis is a rare diagnosis and CA is especially diagnosed very late in the process. The limited study population and late referral to tertiary centers makes this a challenging endeavor. The study is provided with relevant intro, patient characteristics, statistical analyses and references. Additional studies with increased enrollment is needed. In the interim, smaller studies like these with an understanding of its limitations are always helpful to advance the understanding of this rare diagnosis.

**We thank this reviewer for these nice comments**
