# Supplementary material for: Catheter ablation of atrial arrhythmias in cardiac amyloidosis: Impact on heart failure and mortality
Source: PLoS One. 2024 Apr 5;19(4):e0301753. doi: 10.1371/journal.pone.0301753 (PMC10997066; doi:10.1371/journal.pone.0301753)
Supplement: Attachment — Submitted filename: answer to the editos comments.doc [file pone.0301753.s006.doc]

PONE-D-22-29848R3

Catheter Ablation of Atrial Arrhythmias in Cardiac Amyloidosis. Impact on Heart Failure and Mortality

PLOS ONE

Answers to the editor’s comments

I do not completely agree with conclusions of the results as stated by authors

In the results section

When results are not significant, not sure we can there is a positive trend ( especially when the total N is also very low), So not sure if we can come to this conclusion

-Ex” Lef atrial volume and Sys pulm artery pressure: P is 0.6 and 0.26 with only minimal change in pulm artery pressure?). So not sure if we can come to this conclusion

-There was no significant difference in HF hospitalizations ( p=0.1) and all cause mortality (p=0.2)when comparing with AA recurrence.

-Even in patients with permanent AA, p is not significant ( so not sure we can say trend towards higher mortality)

So in conclusion, I agree we can say may be NYHA class and BNP numbers are improving, but rest of the parameters are not showing a significant difference, so not sure we can say positive trend repeatedly despite P value not being significant

Answer: We agree this was too optimistic. We have changed most of these sentences

“left atrial dimension and pulmonary arterial pressure decreased after ablation in patients in sinus rhythm although not significantly”. (page 10, line 1)

“All-cause mortality was not associated with AA recurrence (5/17 wo AA recurrence vs 7/14 with AA recurrence, p=0.2), and mortality in patients with permanent AA (5/10) was not significantly higher compared to patients remaining in SR (7/21) (p=0.3)”. page 10 line 11

However we decided to keep unchanged:

“There was a trend toward less hospitalizations for HF in patients remaining in SR over the follow-up period (2/21) compared to patients with AA recurrence (5/10) (p=0.1).” (p=0.1 may be considered as a trend in such a limited population) page 10 line 7

“All 3 patients with deaths caused by intractable HF had AA recurrence compared to 11 out of the remaining 28 long-term survivors (or dead but not related to HF) (p=0.04)”. page 10 line 9

Paper still has some grammatical errors which need to be corrected ( please see attached file with comments)

We have modified the manuscript accordingly

In the introduction: authors mention that rate control is challenging due to negative inotropic and chronotropic effects of drugs ( can we explain more about this ?) and can we explain why only amio is the drug used

This has been more explained : “Since cardiac output in CA is highly dependent on heart rate because of altered diastolic filling and since CA is associated with frequent conduction disturbances, rate control is challenging because of the vasodilator, potentially toxic, negative inotropic, dromotropic and especially chronotropic effects of drugs (2, 6). Due to before mentioned reasons, amiodarone is the only anti-arrhythmic drug used in this population, but it has been shown to be ineffective in maintaining SR in majority of the cases (10).” Page 3 line 17

Discussion section needs to report results of the paper more in comparison with other ( please see attached paper with comments)answer to the editor’s comments

A chapter dedicated to the comparison with other studies was already included rather early in the discussion, so we feel this part is enough complete and rather well placed in the discussion part
